# Low penicillin susceptibility in *Streptococcus mitis/oralis* from bloodstream infections in pediatric populations

**DOI:** 10.1128/spectrum.01350-24

**Published:** 2024-10-14

**Authors:** Jian-Ming Zhou, Cai-Yu Wu, Jian-Ping Li, Yi-Hua Ye, Chao Fang, Ming-Ming Zhou

**Affiliations:** 1 Department of Clinical Laboratory, Children’s Hospital, Zhejiang University School of Medicine, National Clinical Research Center for Child Health, National Children’s Regional Medical Center, Hangzhou, Zhejiang, China; 2 Department of Clinical Laboratory, Beidahuang Industry Group General Hospital, Harbin, Heilongjiang, China; Ann & Robert H. Lurie Children's Hospital of Chicago, Chicago, Illinois, USA

**Keywords:** *Streptococcus mitis*, bloodstream infections, susceptibility, multi-drug-resistant, children

## Abstract

*Streptococcus mitis/oralis* can cause invasive diseases, including bloodstream infections. However, existing research primarily focuses on specific populations, and limited studies have been conducted on the prevalence of bloodstream infection caused by *S. mitis/oralis* across the entire pediatric population. Therefore, clinical data of *S. mitis/oralis* isolated from blood samples at Children’s Hospital, Zhejiang University School of Medicine, during the period 2019–2023 were collected retrospectively to provide a comprehensive understanding of the clinical characteristics and drug resistance patterns associated with bloodstream infections caused by *S. mitis/oralis* in pediatric populations. There were 57 (43.5%) instances of contamination across various departments, indicating a relatively dispersed pattern. Bloodstream infections caused by *S. mitis/oralis* are notably prevalent among pediatric patients with hematological diseases and tumors. The susceptibility rates of the 74 *S. mitis/oralis* isolates to different antibiotics were as follows: penicillin (23%), ceftriaxone (74.3%), levofloxacin (86.5%), chloramphenicol (89.2%), erythromycin (27%), clindamycin (67.6%), linezolid (100%), and vancomycin (100%). Notably, 21.6% of the isolates exhibited multi-drug resistance (MDR). The predominant mode of MDR in *S. mitis/oralis* infections was identified as resistance to β-lactams, erythromycin, and clindamycin. The observed low susceptibility rate to penicillin, coupled with the emergence of MDR strains, underscores the imperative for continuous monitoring of the evolving antimicrobial resistance in *S. mitis/oralis*.

**IMPORTANCE:**

Existing research primarily focuses on specific populations, such as those with hematopathy or tumors, who experience *Streptococcus mitis/oralis* bacteremia. Limited studies have been conducted on the prevalence of bloodstream infections caused by *S. mitis/oralis* across the entire pediatric population. It was found that the contamination rate of *S. mitis/oralis* isolated from blood cultures was notably high in our study. Therefore, this study evaluated the clinical characteristics and drug resistance patterns of bloodstream infections caused by *S. mitis/oralis* across the entire pediatric populations, explicitly excluding cases of blood culture contamination. The observed low susceptibility rate to penicillin, coupled with the emergence of multi-drug-resistant strains, underscores the imperative for continuous monitoring of the evolving antimicrobial resistance in *S. mitis/oralis*.

## INTRODUCTION

*Streptococcus mitis/oralis*, which falls under the category of viridans group streptococci (VGS), encompasses species including *S. mitis* and *S. oralis* (1). While *S. mitis/oralis* are typically found as colonized bacteria in the human oral cavity, digestive tract, and female reproductive tract, they can cause invasive diseases such as endocarditis, meningitis, and pneumonia when the immune function is compromised (2). The prevalence of VGS infection has shown an upward trend in recent years, particularly among patients with neutropenia (3). In the case of children with malignant tumors complicated with VGS, *S. mitis/oralis* has been found to account for more than 90% of cases, and patients infected with *S. mitis/oralis* were more likely to develop moderate and severe clinical diseases than other streptococci (4). However, existing research primarily focuses on specific populations, such as those with hematopathy or tumors, who experience *S. mitis/oralis* bacteremia. Limited studies have been conducted on the prevalence of bloodstream infection caused by *S. mitis/oralis* across the entire pediatric population.

There are limited data available showing antibiotic susceptibility over time for VGS (5). The increase in the proportion of penicillin-resistant *S. mitis/oralis* strains makes the treatment of such infections more complicated (6, 7). Consequently, there is an urgent need for antimicrobial susceptibility testing (AST) data to guide antibacterial therapy. Therefore, *S. mitis/oralis* isolates from bloodstream in children were collected and analyzed in this study to provide comprehensive and valuable insights into the prevalence and antimicrobial susceptibility in pediatric populations.

## MATERIALS AND METHODS

### Collection of isolated strains and clinical data

The clinical data of patients with *S. mitis/oralis* isolated from blood samples at Children’s Hospital, Zhejiang University School of Medicine, during the period 2019–2023 were collected retrospectively. Positive blood cultures were categorized as true positives or contaminated cultures based on the published criteria of Weinstein *et al.* (8). The categorical decision was made after the following factors were taken into account: the patient’s clinical history, physical findings, body temperature, laboratory test results (time to positivity, leukocyte count and differential cell counts, C-reactive protein, procalcitonin), clinical course, and response to therapy. Bloodstream infection was defined as the presence of true-positive blood culture, along with clinical signs and symptoms of infection (9). Cases not meeting the definition of infection case were judged as cases of contamination and not included in the analysis. The bloodstream infection cases were classified based on the following age categories: neonatal period (0–28 days), infantile period (29–364 days), toddler/early childhood (1 to <6 years), middle childhood (6 to <12 years), and adolescence (12–18 years).

### Bacterial culture and identification

The blood samples were cultured by an automatic blood culture system (BACTEC FX, BD). For positive blood cultures, subculturing was performed on Columbia blood agar plates. Then the bacteria strains were identified with Matrix-Assisted Laser Desorption/Ionization Time of Flight Mass Spectrometry (Bruker, Germany). Duplicate strains from the same patient, same sample source, and same site were eliminated.

### Antimicrobial susceptibility testing

AST was performed according to the Clinical and Laboratory Standards Institute (CLSI) methodology. The disk diffusion method was used to measure the diameter of the zone of inhibition for ceftriaxone (30 µg, OXOID, UK), erythromycin (15 µg, OXOID, UK), clindamycin (2 µg, OXOID, UK), levofloxacin (5 µg, OXOID, UK), chloramphenicol (30 µg, OXOID, UK), linezolid (30 µg, OXOID, UK), and vancomycin (30 µg, OXOID, UK). The E-test was used to determine the minimal inhibitory concentration (MIC) of penicillin (BIO-KONT, China). *Streptococcus pneumoniae* ATCC49619 was used as the quality control strain for both E-test and disk diffusion. The expected quality control ranges of ATCC49619 and the interpretation of antimicrobial susceptibility results follow the guidelines set by the CLSI M100 33ED (10). Multi-drug resistance (MDR) was defined as being resistant to three or more different classes of antimicrobial agents.

## RESULTS

### General information and clinical characteristics

A total of 131 strains of *S. mitis/oralis* were identified from blood samples collected between 2019 and 2023, of which 57 cases (43.5%) were excluded due to contamination. Consequently, a total of 74 cases were included in the present study. The distribution of the 74 cases isolated from 2019 to 2023 was 10.8, 8.1, 29.7, 35.2, and 16.2%, respectively. The gender distribution consisted of 40 males (54.1%) and 34 females (45.9%). The age distribution primarily encompassed toddler/early childhood (51.3%) as well as middle childhood (24.3%), with a median age of 3 years and an interquartile range of 1–6 years. The main clinical departments involved in the study were hematology (52.7%), oncologic surgery (9.4%), neonatal intensive care unit (NICU, 5.4%), and cardiology (5.4%). A total of 49 patients were diagnosed with agranulocytosis (66.2%) (Table 1).

**TABLE 1:** Clinical characteristics of patients with bloodstream infection caused by *S. mitis/oralis*

Clinical characteristics	Cases	Rate (%)
Year		
2019	8	10.8
2020	6	8.1
2021	22	29.7
2022	26	35.2
2023	12	16.2
Gender		
Male	40	54.1
Female	34	45.9
Age		
Neonatal period	2	2.7
Infantile period	11	14.9
Toddler/early childhood	38	51.3
Middle childhood	18	24.3
Adolescence	5	6.8
Clinical departments		
Hematology	39	52.7
Oncological surgery	7	9.4
NICU	4	5.4
Cardiology	4	5.4
Others^*a*^	20	35.1
Neutrophils		
Agranulocytosis^*b*^	49	66.2
Non*-*agranulocytosis	25	33.8

^*a*^ Contains 11 clinical departments (cardiac surgery intensive care unit, outpatient department, emergency department, respiratory medicine department, general surgery department, cardiac surgery department, neonatology department, pediatric intensive care unit, surgical intensive care unit, rheumatology and immunology department, nephrology department).

^*b*^ Neutrophil absolute value <0.5 * 10^9^/L.

### Antimicrobial susceptibility testing

Among these patients, 74 strains of *S. mitis/oralis* exhibited 100% susceptibility to linezolid and vancomycin. The rates of susceptibility to penicillin, ceftriaxone, levofloxacin, chloramphenicol, erythromycin, and clindamycin were found to be 23, 74.3, 67.6, 86.5, 27, and 67.6%, respectively. Penicillin intermediate resistance was found to be 50%, as given in Table 2. Out of the overall 74 strains, 16 (21.6%) strains were identified as MDR. The predominant mode of MDR was identified as resistance to β-lactams, erythromycin, and clindamycin (Table 3).

**TABLE 2:** Antimicrobial susceptibility of *S. mitis/oralis* isolates

Antimicrobial	susceptible		intermediate		resistant	
	*n*	%	*n*	%	*n*	%
Penicillin	17	23	37	50	20	27
Ceftriaxone	55	74.3	4	5.4	15	20.3
Erythromycin	20	27	17	23	37	50
Clindamycin	50	67.6	0	0	24	32.4
Levofloxacin	64	86.5	1	1.4	9	12.1
Chloramphenicol	66	89.2	4	5.4	4	5.4
Linezolid	74	100	0	0	0	0
Vancomycin	74	100	0	0	0	0

**TABLE 3:** The MDR phenotype of *S. mitis/oralis* isolates

MDR phenotype	*n*	%
β-Lactams–erythromycin–clindamycin	7	43.8
Erythromycin–clindamycin–levofloxacin	3	18.7
β-Lactams–erythromycin–chloramphenicol	3	18.7
β-Lactams–erythromycin–clindamycin–levofloxacin	2	12.5
β-Lactams–erythromycin–clindamycin–levofloxacin–chloramphenicol	1	6.3

## DISCUSSION

Since the initial report of a neonatal meningitis case caused by *S. mitis* in 1985 (11), the infection caused by this bacterium has garnered increasing attention, as evidenced by successive reports on *S. mitis* infection in children in recent years (2, 12, 13). The present study reveals a significant rise in the number of cases of bloodstream infection caused by *S. mitis/oralis* in 2021 and 2022, indicating a growing incidence of such infections that warrants significant clinical concern.

Blood culture contamination has been recognized as an important quality problem for decades. This contamination arises from the introduction of extraneous organisms, such as those from the skin or environment, into the blood sample collected for culture. Such contamination results can result in inaccurate patient diagnoses, improper follow-up, and unwarranted treatments (14). *S. mitis/oralis*, although capable of causing bloodstream infections through oral and dental routes (15), can also contribute to blood culture contamination. Therefore, it is crucial to thoroughly assess the clinical condition to accurately interpret bloodstream infections attributed to *S. mitis/oralis*. Consequently, cases of contamination were meticulously excluded from this study, ensuring that only genuine infection cases were included. This methodological rigor represents a novel aspect of the study’s design. Notably, the study identified contamination in up to 43.5% of cases, a rate higher than reported in previous studies, which are based on a smaller sample size (8). In the present study, cases with contamination of *S. mitis/oralis* were dispersed across 11 different departments. In contrast, instances of bloodstream infections caused by *S. mitis/oralis* were predominantly observed in patients with agranulocytosis, particularly within hematology and oncology surgery departments, aligning with previous reports (2, 4). It is worth noting that all instances of *S. mitis/oralis* identified within the population with agranulocytosis in this study were classified as infections according to our established criteria. Furthermore, it is noteworthy that children with low immunity, specifically those in NICU, are susceptible to bloodstream infections caused by *S. mitis/oralis*. Additionally, children with endocarditis in cardiology departments also require attention regarding the risk of bloodstream infections caused by *S. mitis/oralis*. The findings of this study indicate that the age group most affected by bloodstream infections caused by *S. mitis/oralis* is between 1 and <6 years old, with a median age of 3 years. This aligns with previous reports indicating a median age of 27 months (2), underscoring the critical need for vigilance and attention toward young children.

Furthermore, it is noteworthy that the emergence of penicillin resistance in VGS has led several countries to discontinue the use of penicillin as their empirical treatment (16). In this study, the drug resistance rate of *S. mitis/oralis* isolates to penicillin was determined to be 27%, which is marginally lower than the rate observed in Turkish children with blood infection caused by *S. mitis*/*oralis* (45.2%) (2), but substantially higher than the drug resistance rate of *S. mitis*/*oralis* in the national surveillance in the Netherlands (6%) (17) and the drug resistance rate of all VGS (contains *S. mitis*/*oralis* and other VGS) in the China Antimicrobial Surveillance Network (CHINET) (5.7–8.5% in 2015–2021) (18). On the one hand, the variation in drug resistance could be due to the inclusion of *S. mitis/oralis* and other streptococci groups in the CHINET study, leading to disparities in drug resistance. On the other hand, previous reports have indicated a higher drug resistance rate among children compared with adults (19). Furthermore, our study found that 50% of *S. mitis/oralis* isolates had intermediate resistance to penicillin, with only 23% being susceptible, suggesting that penicillin is no longer suitable for empirical treatment of bloodstream infections caused by *S. mitis/oralis* in children within our hospital. Instead, third-generation cephalosporins and vancomycin can be considered for cases of penicillin-intermediate or penicillin-resistant *Streptococcus* bloodstream infection (20). The present study also revealed that the resistance rate of *S. mitis/oralis* isolates to ceftriaxone was 20.3%, which was lower than the rate observed in children with *S. mitis*/*oralis* bloodstream infection in Turkey (39.5%) (2), but higher than the drug resistance rate reported in national surveillance in the Netherlands (7.6%) (17) and drug resistance rate of all VGS (contains *S. mitis*/*oralis* and other VGS) in CHINET in 2015–2021 (6.9–13.7%) (18). Considering the escalating drug resistance rate of ceftriaxone, it is imperative to choose appropriate treatment based on the results of AST. Multiple studies have demonstrated the absence of resistance to linezolid and vancomycin among strains of the *S. mitis* group (2, 18). In line with these findings, all 74 strains of *S. mitis/oralis* exhibited complete susceptibility to linezolid and vancomycin. Given the low susceptibility rates of penicillin and ceftriaxone, vancomycin may be considered as the empiric therapy for bloodstream infections caused by *S. mitis/oralis*, particularly in immunocompromised populations, such as those with agranulocytosis.

There was a limited number of studies conducted on MDR strains of *S. mitis/oralis*. In 2019, a case involving a 7-month-old infant with a retropharyngeal abscess was reported, and the MDR phenotype exhibited resistance to cephalosporins, macrolides, and fluoroquinolones (19). Additionally, another autopsy case of infective endocarditis caused by MDR *S. mitis* was documented, where the *S. mitis* strain isolated from a blood culture demonstrated widespread resistance to penicillin, cephalosporins, carbapenem, macrolides, and fluoroquinolones (21). Within the scope of this study, it was determined that 16 strains (21.6%) of *S. mitis/oralis* were classified as MDR strains, with the predominant MDR pattern being resistant to β-lactam, erythromycin, and clindamycin. This finding suggests that the occurrence of MDR strains within *S. mitis/oralis* is not uncommon, highlighting the need for clinical awareness. Consequently, clinical treatment should be determined based on the results of AST.

## CONCLUSION AND LIMITATIONS

As previously discussed, the contamination rate of *S. mitis/oralis* isolated from blood cultures was notably high. This study aimed to assess the clinical characteristics and antimicrobial resistance patterns of bloodstream infections caused by *S. mitis/oralis* in the entire pediatric population, explicitly excluding instances of blood culture contamination. Bloodstream infections attributed to *S. mitis/oralis* are particularly prevalent among children with hematological malignancies and tumors. Notably, *S. mitis/oralis* isolated from pediatric bloodstream infections demonstrates low susceptibility to penicillin, rendering it unsuitable for empirical treatment. Additionally, there is a high resistance rate to ceftriaxone, necessitating its selection based on AST results. In critically ill pediatric patients, linezolid and vancomycin may serve as effective therapeutic options. The emergence of MDR strains underscores the imperative for clinical vigilance regarding their drug resistance profiles.

However, there are two main limitations to this study. Following the exclusion of contaminated samples, the study ultimately included only 74 confirmed cases of *S. mitis/oralis* bloodstream infection, which is a relatively small sample size. Future research should prioritize the expansion of the sample size to address this limitation and yield more robust and reliable evidence for clinical diagnosis and treatment. Additionally, AST primarily employed the disk diffusion method, which does not facilitate a comprehensive analysis of the MIC. A more detailed examination of MIC could offer valuable insights pertinent to treatment options in clinical settings.
